# Psychosocial outcomes in Chinese survivors of pediatric cancers or bone marrow failure disorders: A single-center study

**DOI:** 10.1371/journal.pone.0279112

**Published:** 2022-12-13

**Authors:** Jiaoyang Cai, Yin Ting Cheung, Phillip Lung Wai Au-Doung, Wenting Hu, Yijin Gao, Hua Zhang, Mingjing Ji, Shuhong Shen, Jing Chen, Jingyan Tang

**Affiliations:** 1 Department of Hematology/Oncology, Shanghai Children’s Medical Center, Shanghai Jiao Tong University School of Medicine, Key Laboratory of Pediatric Hematology and Oncology of China Ministry of Health, Shanghai, China; 2 Faculty of Medicine, School of Pharmacy, The Chinese University of Hong Kong, Hong Kong SAR, China; Istanbul University-Cerrahpaşa, Cerrahpaşa Faculty of Medicine, TURKEY

## Abstract

**Background/Objectives:**

Most of the studies on functional outcomes in pediatric survivors of cancers and bone marrow failure disorders have been conducted in North American, European, and Oceanian populations, with few studies having been performed in China. The objective of this study was to evaluate psychosocial outcomes in a cohort of Chinese pediatric survivors diagnosed with cancer or conditions requiring hematopoietic stem cell transplantation (HSCT), and to identify clinical and behavioral factors associated with adverse psychosocial outcomes.

**Methods:**

This was a cross-sectional survey study. We recruited pediatric survivors of cancer or inherited disorder requiring HSCT at ≤18 years old and were ≥6 months post-treatment. Parents completed the St. Jude Children’s Research Hospital After Completion of Therapy questionnaire to report their child’s emotional functioning, social functioning, attention/concentration and behavior. Multivariable general linear modeling was used to identify clinical, treatment and behavioral factors associated with psychosocial outcomes, adjusting for sex, age and cancer diagnoses.

**Results:**

Ninety-five pediatric survivors were recruited (62.1% male; mean [standard deviation] age 9.7 [3.4] years; 4.1 [2.6] years post-diagnosis). They were diagnosed with bone marrow failure disorders (23.2%), hematological malignancies (45.3%) or solid tumors (23.2%). Compared with survivors with no current health problems, those with more than one current health problem performed worse in emotional functioning (Estimate = 2.42, SE = 0.88, *P* = 0.008) and social functioning (Estimate = 2.90, SE = 1.64, *P* = 0.03). Higher pain interference was significantly associated with worse emotional functioning (Estimate = 0.19, SE = 0.08, *P* = 0.03) and attention functioning (Estimate = 0.26, SE = 0.11, *P* = 0.03). Compared with survivors who reported less sleep problems, those who had more sleep problems demonstrated poorer emotional functioning (Estimate = 0.30, SE = 0.08, *P* = 0.001). Survivors who had a longer duration of screen usage per day reported more impairment on attention and behavior functioning than those who had a shorter duration of screen usage per day (both *P*<0.5).

**Conclusion:**

Survivors who were diagnosed at a younger age or had unaddressed/untreated health problems may require additional psychological evaluation. The implementation of psychosocial assessments during routine long-term follow-up care may help to identify high-risk patients during the early phase of survivorship. Rehabilitation interventions should address modifiable behavioral factors (e.g. sleep habits, screen time and chronic pain).

## Introduction

Due to advances in early detection and effective treatments, the survival rates of pediatric survivors of cancer and bone marrow failure disorders have improved dramatically over the past few decades [1–3]. In most developed countries, approximately 80% of pediatric cancer patients who receive contemporary treatment regimens (comprising surgery, radiotherapy, chemotherapy, hematopoietic stem cell transplantation [HSCT] and/or targeted therapies) now survive beyond 5 years after diagnosis [4]. However, children who survived intensive cancer treatments and HSCT often suffered from treatment-related late effects and cancer-related symptoms [5,6].

Other than chronic health conditions, children with cancer and bone marrow failure disorders also experience symptoms of cognitive deficits, psychological distress, and pain and behavioral problems [7–10]. These symptoms can emerge during the active treatment phase and persist until survivorship [7–11]. For example, one large study in the United Kingdom reported that neurodevelopmental disorders and health conditions, such as migraines and atopic conditions, were strongly associated with mental health behaviors in children with long-term illnesses [12]. Specifically, while most pediatric cancer survivors are well at long-term follow-up, they have been found to have a higher frequency of persistent behavioral problems and impairments than the general population [8,9,11]. Children with hematological or bone marrow failure disorders requiring HSCT might demonstrate depression, anxiety, sleep disruption and adjustment disorders due to prolonged periods of hospitalization and isolation [13,14]. During the recovery or survivorship phase, children with such chronic diseases also face challenges in school from frequent absenteeism, difficulties meeting academic expectations, and disrupted relationships with their peers [15,16]. These behavioral symptoms might negatively impact survivors’ psychosocial and functional outcomes as they transition to young adulthood [17–19].

To date, most of the studies on functional outcomes in pediatric survivors of cancers and bone marrow failure disorders have been conducted in North American, European, and Oceanian populations, with few studies having been performed in China. Moreover, evidence from survivorship studies conducted in Western populations cannot be extrapolated to Chinese populations, due to the differences between these populations’ cultural-related behaviors and the environmental factors to which they are exposed [20,21]. In mainland China, the well-being of children with chronic diseases might be affected by region and culture-specific factors such as living space, residential area (urban versus rural), availability of domestic help, and social benefits/ support by the municipality [22]. Ethnic and sociocultural constructs may therefore cause treatments to differentially affect perceptive and psychosocial development in Asian and Western pediatric survivors of cancers and bone marrow failure disorders.

The objective of this study was to evaluate psychosocial outcomes in a cohort of Chinese pediatric survivors diagnosed with cancer or bone marrow failure disorders requiring HSCT, and to identify clinical and behavioral factors associated with poor outcomes in this population.

## Methods

This was a cross-sectional study with prospective data collection conducted at the Long-Term Follow-up (LTFU) Clinic at Shanghai Children’s Medical Center (SCMC). The SCMC is a major pediatric oncology center in the Chinese Children’s Cancer Group (CCCG) [21,23,24]. This study was approved by the Research Ethics Boards at Shanghai Children’s Medical Center, and written informed consent was obtained from all participants.

### Study population

The participants were recruited from the LTFU clinic from January 2020 to June 2021 by consecutive sampling. Survivors were eligible for this study if they had been diagnosed with cancer or an inherited disorder requiring HSCT before the age of 18 years and was more than 6 months post-treatment at the time of assessment. In addition, the survivors’ parents were required to be able to speak Mandarin and read Chinese. If both parents were present, one parent per family unit, preferably the primary caregiver who interacted with the child most frequently, was recruited.

### Study procedure

The clinical information on survivors’ cancer diagnosis, treatment modalities, age at diagnosis, and time since diagnosis were abstracted from medical records. Each parent completed a structured questionnaire in paper format. The questionnaire collected the survivors’ sociodemographic information (living arrangements, highest education attainment and insurance access) and health behaviors (i.e. sleep problems, physical activity level and screen time). It also collected information on the survivors’ current health status, which included a brief pain assessment and a list of 22 current health problems (S1 File).

### Study outcomes

The four outcomes of interest were based on the parents’ measures of their child’s emotional functioning, social functioning, attention/concentration and behavior. These measures were captured using the St. Jude Children’s Research Hospital After Completion of Therapy (ACT) questionnaire [25]. Permission was obtained from the developers to translate the questionnaire into Simplified Chinese, and this version of the questionnaire has been used since 2019 by pediatric oncologists and hematologists at the LTFU clinic of SCMC to evaluate the health status of pediatric survivors. The Simplified Chinese version of the ACT Questionnaire consists of questions on four scales: an emotional functioning scale, a social functioning scale, an attention/concentration scale and a child behavior scale.

The emotional functioning scale consists of six items (score range: 1 to 30 points) that evaluate whether a child “cannot stop feeling sad,” always feels “everything in his/her life goes wrong,” that “he/she cannot do anything right,” “lonely,” “sad” or that “his/her life is bad.” The social functioning scale consists of seven items (score range: 1 to 35 points) examining a child’s relationship with other children (e.g. whether he/she feels accepted, makes friends, helps others and talks to other children). The attention/concentration scale consists of six items (score range: 1 to 30 points) that assess a child’s ability to pay attention to detail and whether he/she has problems concentrating on his/her work, paying attention for prolonged periods, finishing tasks or keeping track of what he/she is doing. Parents rate their child’s functioning on these three scales using a Likert scale of 1 (never) to 5 (almost always). The scores for the items on each of these three scales are summed to generate a total score for each scale, with a higher score indicative of worse functioning.

The child behavior scale consists of 23 items adapted from the Behavior Problems Index [26]. It evaluates a child’s attention problems, internalizing problems (anxiety and depressive symptoms, mood swings and withdrawn behaviors) and externalizing problems (aggression, misbehavior, conduct disorder and strong temper). Parents rate the frequency of these symptoms in their child on a Likert scale from 1 (not true) to 3 (often true). The total possible score on this scale ranges from 1 to 69 points, with a higher score indicative of more behavioral problems.

### Predictors

The following clinically relevant predictors were determined *a priori* as studies have suggested that they can affect psychosocial functioning in survivors [9–11,17,18,27–29]. The clinical predictors were age at diagnosis, time since diagnosis and diagnosis (hematological cancers *versus* solid tumors *versus* bone marrow failure disorders) [8,27,30]. As there is emerging evidence supporting the impact of chronic health problems and a compromised health status on functional outcomes in survivors of childhood cancer [27,31–34], our analysis also included the presence of current health problems/symptoms as one of the clinical predictors. This factor was assessed based on an item in the ACT questionnaire, which required parents to indicate if the child presented with any of the 22 health problems or symptoms listed in S1 File within the past month. Subsequently, the attending physician would verify the response by conducting further physical assessments or soliciting more information from the parent regarding the health problem.

The treatment factors were treatment modality (surgery, chemotherapy, radiation and/or HSCT) and time since end of treatment [8,29,30,35]. The behavioral factors were physical activity (≤ 1 day per week *versus* > 1 day per week), screen time (≤ 3 h per day *versus* > 3 h per day), sleep problems and pain interference [7,10,36–38].

### Statistical analysis

Descriptive statistics were used to summarize the study outcomes and all of the covariates. The categorical variables are presented as absolute frequencies and percentages, whereas the continuous variables are presented as means and standard deviations (SDs).

The categorical variables were subjected to a univariate analysis using an independent *t*-test or a one-way analysis of variance, and the continuous variables were analyzed via Pearson’s correlation test to compare differences in study outcomes between survivors in clinically relevant subgroups. The clinically relevant subgroups referred to the clinical, treatment, and behavioral factors listed in the previous section. The comparisons were adjusted for false discovery rate [39]. An exploratory analysis was conducted to compare outcomes among survivors who reported health problems/ symptoms that demonstrate strong level of evidence in their associations with psychosocial functioning in the literature (e.g. chronic pain, cardiac symptoms, and fatigue) [7,9,10,32,36,40–42].

A forward stepwise linear regression (*P* < 0.10 to enter, *P* > 0.15 to remove) was used to identify factors associated with functional outcomes, with sex, age and cancer diagnoses as fixed factors. Model selection was conducted using the Akaike information criterion. Visual graphical inspection of the diagnostic plots was used to examine the normality of residues and homogeneity of residuals variance. Unstandardized point estimates (Est) and standard errors (SE) were calculated to quantify the effect size of associations.

All of the statistical analyses were performed using SPSS software, version 20 (Armonk, NY: IBM Corp). All of the tests were two-tailed, and the results were considered statistically significant at *p* < 0.05.

## Results

Ninety-five pediatric survivors participated in this study. Their demographic and clinical characteristics are summarized in Table 1. The majority were male (*n* = 59, 62.1%). The mean [SD] age of the survivors at assessment was 9.7 [3.4] years, which was an average of 4.1 [2.6] years after diagnosis. The majority of the survivors were attending school (*n* = 86, 90.5%) at the time of assessment. Being physically weak (*n* = 5), incomplete vaccination (*n* = 1), learning problems (*n* = 1) and currently still under school age (*n* = 2) were the main reasons for not attending school.

**Table 1:** Demographic, clinical, and treatment characteristics of survivors (n = 95).

Characteristics	n (%)
**Sex of survivor**	
Male	59 (62.1)
Female	36 (37.9)
**Current age (years)**	
Mean ± SD	9.7 (3.4)
≤5	6 (6.3)
5–9	44 (46.3)
10–14	36 (37.9)
≥15	9 (9.5)
**Education attainment**	
**Currently not attending school**	**9 (9.5%)**
**Currently attending school**	**86 (90.5%)**
Elementary school or below	66
Middle school	17
High school or above	3
**Age at diagnosis (years)**	
Mean ± (SD)	5.0 (3.5)
≤5	49 (51.6)
5–9	31 (32.6)
10–15	15 (15.8)
**Time since diagnosis (years)**	
Mean ± SD	4.1 (2.6)
<5	55 (57.9)
5–9	35 (36.8)
≥10	5 (5.3)
**Clinical diagnosis**	
**Bone marrow failure disorders**	**22 (23.2)**
Aplastic anemia	11
Langerhans cell histiocytosis	8
Thalassemia	2
Fanconi anemia	1
**Hematological malignancy**	**43 (45.3)**
Acute lymphoblastic leukemia	19
Lymphoma	15
Acute myeloid leukemia	6
Myelodysplastic syndromes	2
Chronic myeloid leukemia	1
**Solid tumors**	**22 (23.2)**
Wilms tumor	6
Neuroblastoma	5
Sarcoma	5
Hepatoblastoma	4
Germ cell tumor	2
Others*	**8 (8.4)**
**Treatment**	
Surgery only	2 (2.1)
Chemotherapy only	28 (29.5)
Surgery and chemotherapy	22 (23.2)
Radiotherapy, surgery and chemotherapy	12 (12.6)
Hematopoietic stem cell transplantation	31 (32.6)
**Chemotherapy treatment characteristics**	
Any	70 (73.7)
Corticosteroids	43 (45.3)
Anthracyclines/ anthraquinone	54 (56.8)
Antimetabolics	46 (48.4)
Alkylating agents	55 (57.9)
Plant alkaloids	58 (61.1)
Topoisomerase inhibitors	37 (38.6)
**Time since end of treatment (years)**	
Mean ± (SD)	2.7 (2.3)
≤5	74 (82.2)
5–9	14 (15.6)
10–15	2 (2.2)
**Current health problem/ symptom ^**	
None	43 (47.8)
1 problem	22 (24.4)
> 1 problem	25 (27.8)
Physical activity ^	
Exercise ≥ 2 days per week	74 (77.9)
Exercise ≤ 1 day per week	16 (16.8)
Screen time Ɨ	
≤ 3 hours per day	67 (70.5)
> 3 hours per day	26 (27.5)
Respondent’s relationship with survivor ^§^	
Father	32 (33.7)
Mother	51 (53.7)

*Other diagnoses: Adrenoleukodystrophy (n = 2), mucopolysaccharidosis II (n = 3), *Hyper*-*IgM (n = 3)*.

^ Missing (n = 5). The rates of the specific health problems or symptoms are presented in S1 File.

Ɨ Missing (n = 2). Screen time includes time spent on the television, computers, tablets and smartphones.

^§^ Missing (n = 12). The parent who spent the most time providing direct care to the child was encouraged to be the respondent.

The survivors were diagnosed at a mean [SD] age of 5.0 [3.5] years. The most common diagnoses of survivors with a bone marrow failure disorder (*n* = 22, 23.2%) were aplastic anemia (*n* = 11) and Langerhans cell histiocytosis (*n* = 8), while the most common diagnoses of survivors with a hematological malignancy (*n* = 43, 45.3%) were leukemia (*n* = 26) and lymphoma (*n* = 15). The most common diagnoses of survivors with solid tumors (*n* = 22, 23.2%), were Wilms’ tumor (*n* = 6), neuroblastoma (*n =* 5) and sarcoma (*n =* 5). The majority of the survivors had been treated with either chemotherapy-only regimens (*n* = 28, 29.5%) or HSCT (*n* = 31, 32.6%). All of the 31 patients received standard myeloablative busulfan-cyclophosphamide-based conditioning regimens. The majority of the patients (*n* = 74, 82.2%) were within 5 years from completion of treatment.

At the time of assessment, over half of the survivors had at least one parent-reported) health problem or symptom (*n* = 47, 52.2%), which were vision problems, fatigue and/or changes in appetite (S1 File).

A minority of the survivors exercised ≤ 1 day per week (*n* = 16, 16.8%) or had more than 3 hours of screen time per day (*n* = 26, 27.5%).

### Univariate analysis

**Fig 1:**
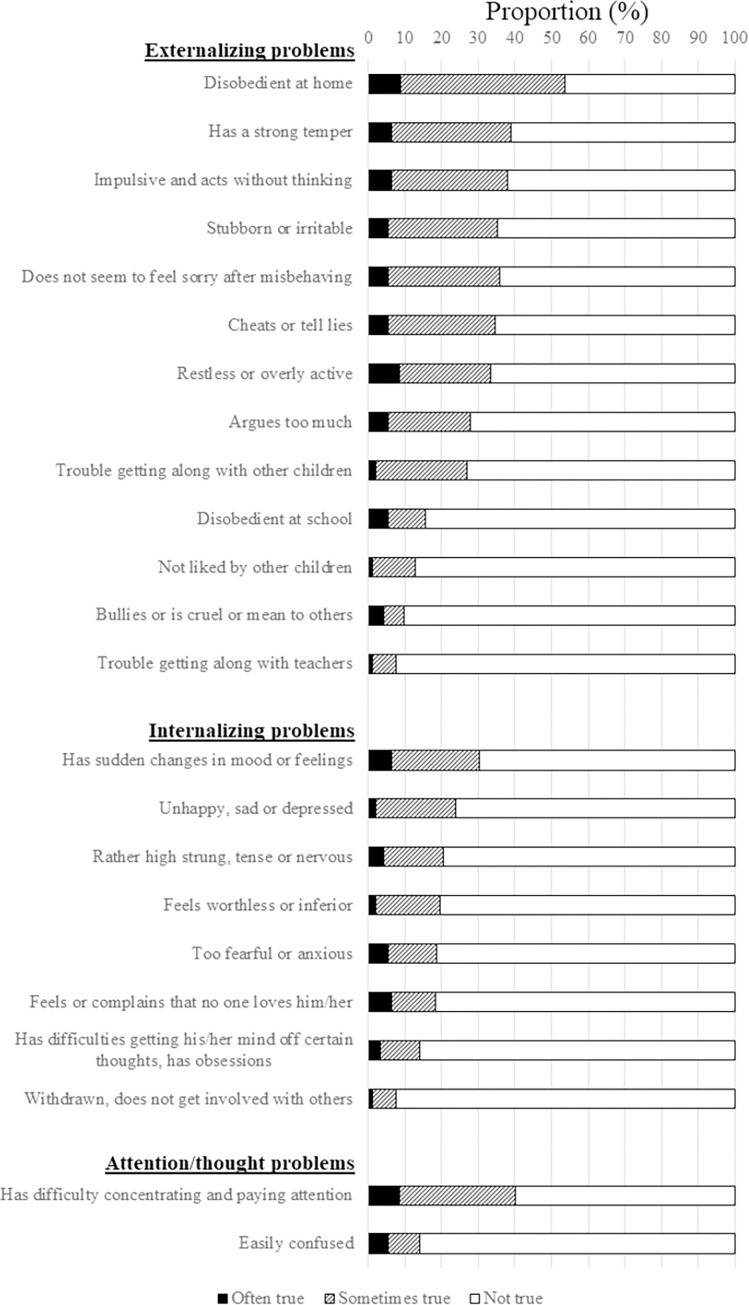
Rates of parent-reported behavioral problems in survivors.

The social functioning (mean [SD], 13.9 [5.6]), emotional functioning (8.8 [3.5]), attention/concentration (13.7 [5.0]) and child behavior (29.6 [7.5]) scores for the cohort are summarized in Table 2. Regarding child behavior, the most common relevant (rated as “often true” or “sometimes true”) parent-reported externalizing problems were misbehavior at home (53.8%), having a strong temper (39.1%) and impulsiveness (38%) (Fig 1), whereas the most common parent-reported internalizing problems were mood swings (30.4%) and depressive symptoms (23.9%).

**Table 2:** Univariate analysis of psychosocial outcomes in survivors across clinically relevant subgroups.

	Psychosocial Impairment Scores*			
	**Social functioning**	**Emotional functioning**	**Attention/ Concentration**	**Child behavior**
Score range	[1 – 35]	[1 – 30]	[1 – 30]	[1 – 69]
All survivors	13.9 ± 5.6	8.8 ± 3.5	13.7 ± 5.0	29.6 ± 7.5
**Demographic factors**				
Age	r_p_ = 0.08 (*P* = 0.57)	r_p_ = -0.09 (*P* = 0.57)	r_p_ = -0.35 **(*P* = 0.01)**	r_p_ = -0.44 **(*P* = 0.005)**
Sex	*P* = 0.82	*P* = 0.73	*P* = 0.64	*P* = 0.99
Male	13.8 ± 5.7	8.9 ± 3.5	13.9 ± 5.4	29.6 ± 6.9
Female	14.2 ± 5.4	8.5 ± 3.5	13.3 ± 4.4	29.6 ± 8.4
**Clinical factors**				
Age at diagnosis (years)^	r_p_ = -0.30 **(*P* = 0.035)**	r_p_ = 0.02 (*P* = 0.86)	r_p_ = -0.13 (*P* = 0.39)	r_p_ = -0.15 (*P* = 0.34)
Time since diagnosis (years)^	r_p_ = 0.30 **(*P* = 0.035)**	r_p_ = -0.09 (*P* = 0.57)	r_p_ = -0.30 **(*P* = 0.02)**	r_p_ = -0.37 **(*P* = 0.005)**
Clinical diagnosis	*P* = 0.96	*P* = 0.57	*P* = 0.57	*P* = 0.19
Bone marrow failure disorders	13.6 ± 6.2	8.7 ± 3.8	13.7 ± 4.5	27.1 ± 5.2
Hematological malignancy	14.1 ± 4.9	9.0 ± 3.7	13.7 ± 5.0	31.0 ± 8.6
Solid tumors	13.7 ± 6.3	7.7 ± 2.5	12.2 ± 4.8	27.4 ± 4.3
Current health problem/ symptoms	*P =* 0.067	***P =* 0.02**	*P =* 0.24	*P =* 0.37
None	12.3 ± 5.3	8.0 ± 3.1	12.8 ± 4.7	28.6 ± 6.4
1 problem	14.6 ± 6.4	8.2 ± 3.4	13.5 ± 5.0	28.6 ± 6.8
> 1 problem	15.6 ± 4.7	10.8 ± 3.7	15.6 ± 5.4	32.1 ± 9.9
**Treatment factors**	*P* = 0.51	*P* = 0.48	*P* = 0.10	*P* = 0.37
Chemotherapy only	13.5 ± 4.8	8.9 3.3	15.0 ± 4.2	30.5 ± 7.5
Surgery and chemotherapy	12.6 ± 4.9	7.5 ± 2.7	11.4 ± 4.8	26.7 ± 3.9
Radiotherapy, surgery and chemotherapy	16.3 ± 7.8	8.8 ± 4.3	12.4 ± 5.7	29.7 ± 6.9
Hematopoietic stem cell transplantation	14.7 ± 5.7	9.7 ± 3.8	14.4 ± 5.5	30.6 ± 9.3
Time since end of treatment (years)^	r_p_ = 0.13 (*P* = 0.34)	r_p_ = 0.008 (*P* = 0.94)	r_p_ = -0.25 (*P* = 0.03)	r_p_ = -0.34 (*P* = 0.01)
**Behavioral factors**				
Physical activity	*P =* 0.075	*P =* 0.35	*P =* 0.83	*P =* 0.97
Exercise ≥ 2 days per week	13.4 ± 5.7	8.4 ± 3.2	13.5 ± 4.7	29.6 ± 7.7
Exercise ≤ 1 days per week	16.4 ± 4.1	10.1 ± 4.3	14.0 ± 6.4	29.2 ± 6.7
Screen time	*P =* 0.10	*P =* 0.73	*P =* 0.12	*P =* 0.10
≤ 3 hours per day	13.3 ± 5.4	8.7 ± 3.4	13.3 ± 5.0	30.9 ± 10.4
> 3 hours per day	15.7 ± 5.7	9.0 ± 3.9	14.8 ± 5.0	29.0 ± 6.0
Sleep problems ^ Ɨ	r_p_ = 0.15 (*P* = 0.34)	r_p_ = 0.40 **(*P* = 0.01)**	r_p_ = 0.08 (*P* = 0.57)	r_p_ = 0.21 (*P* = 0.18)
Pain interference ^ Ɨ	r_p_ = 0.09 (*P* = 0.57)	r_p_ = 0.32 **(*P* = 0.01)**	r_p_ = 0.26 **(*P* = 0.03)**	r_p_ = 0.19 (*P* = 0.19)

* A higher score is indicative of worse functioning.

^ These factors were analyzed as continuous variables. Strength of correlation is presented as the spearman correlation coefficient (r_p_).

Ɨ A higher score is indicative of more sleep problems and pain interference.

All *P*-values are adjusted for false discovery rate.

Younger age was associated with more attention problems (correlation coefficient [r_p_] = -0.35, *P* = 0.01) and behavioral problems (r_p_ = -0.44, *P* = 0.005) (Table 2). Younger age at diagnosis correlated with worse social functioning (correlation coefficient [r_p_] = -0.30, *P* = 0.035). Children with more than one health problem/symptom performed worse in emotional functioning (*P*  =  0.02) than those with only one health problem/symptom or no health problem/symptom. The exploratory analysis suggested that survivors with chronic pain, cardiac, or fatigue-related health problems/ symptoms showed significantly worse social functioning than survivors with other health problems/ symptoms, or no health problem/ symptom (*P* = 0.02) (S2 File).

The survivors who had completed treatment for a longer period of time reported less attention (r_p_ = -0.25, *P* = 0.03) and behavior problems (r_p_ = -0.34, *P* = 0.01) than the survivors who had just completed treatment. There was no statistically significant association between treatment modalities and psychosocial outcomes.

Regarding emotional and behavioral factors, sleep problems were associated with poorer emotional functioning (r_p_ = 0.40, *P* = 0.01). More severe pain interference correlated with more emotional (r_p_ = 0.32, *P* = 0.01) and attention problems (r_p_ = 0.26, *P* = 0.03).

### Multivariable analysis

The results of the multivariable models, adjusted for sex, age and cancer diagnoses, are presented in Table 3. Compared with survivors with no current health problems, those with more than one current health problem performed worse in social functioning (Est = 2.90, SE = 1.64, *P* = 0.03) and emotional functioning (Est = 2.42, SE = 0.88, *P* = 0.008).

**Table 3:** Multivariable analysis of factors associated with psychosocial outcomes.

	Psychosocial Impairment Scores*											
	**Social functioning**			**Emotional functioning**			**Attention/ Concentration**			**Child behavior**		
	Est	SE	*P*	Est	SE	*P*	Est	SE	*P*	Est	SE	*P*
**Clinical factors Ɨ**												
Time since diagnosis^†^	-0.82	0.25	**0.002**	-	-	-	-	-	-	-0.73	0.30	**0.02**
Current health problem												
None	ref			ref			-	-	-	-	-	-
1 problem	1.30	1.53	0.39	0.30	0.88	0.73	-	-	-	-	-	-
> 1 problem	2.90	1.64	**0.03**	2.42	0.88	**0.008**	-	-	-	-	-	-
Treatment factors ^Ɨ^												
Hematopoietic stem cell transplantation (referent)	-	-	-	-	-	-	-	-	-	-	-	-
Chemotherapy only	-	-	-	-	-	-	0.11	1.26	0.92	-	-	-
Surgery and chemotherapy	-	-	-	-	-	-	-3.25	1.38	**0.02**	-	-	-
Radiotherapy, surgery and chemotherapy	-	-	-	-	-	-	-1.86	1.66	0.26	-	-	-
Time since end of treatment (years)^†^	-	-	-	-	-	-	-0.63	0.21	**0.02**	-0.75	0.32	**0.01**
**Behavioral factors Ɨ**												
Physical activity												
Exercise ≤ 1 day per week	ref			-	-	-	-	-	-	-	-	-
Exercise ≥ 2 day per week	3.08	1.73	0.08	-	-	-	-	-	-	-	-	-
Screen time												
≤ 3 hours per day	-	-	-	-	-	-	ref			ref		
> 3 hours per day	-	-	-	-	-	-	2.11	1.15	**0.05**	3.74	1.78	**0.04**
Sleep problems *	0.32	0.14	**0.03**	0.30	0.08	**0.001**	-	-	-	0.37	0.19	0.06
Pain interference *	-	-	-	0.19	0.08	**0.03**	0.26	0.11	**0.03**	-	-	-

Est: Estimate; SE: Standard error.

* A higher score is indicative of worse functioning.

^ All models are adjusted for age, sex and cancer diagnoses.

Ɨ Empty cells (“–”) refer to variables that were eliminated according to the stepwise model selection process.

^†^ Models with time since diagnosis and time since end of treatment were run separately as both variables are highly related to each other (r_p_ = 0.80, *P*<0.0001).

Higher pain interference was significantly associated with worse emotional functioning (Est = 0.19, SE = 0.08, *P* = 0.03) and attention functioning (Est = 0.26, SE = 0.11, *P* = 0.03). Compared with survivors who reported less sleep problems, those who had more sleep problems demonstrated poorer emotional functioning (Est = 0.30, SE = 0.08, *P* = 0.001) and behavior (Est = 0.37, SE = 0.19, *P* = 0.05). Survivors who had a longer duration of screen usage per day reported more impairment on attention (Est = 2.11, SE = 1.15, *P* = 0.05) and behavior (Est = 3.74, SE = 1.78, *P* = 0.04) than those who had a shorter duration of screen usage per day.

## Discussion

Despite continual advances in the treatment of pediatric cancer and bone marrow failure disorders, the screening of psychosocial health in children with chronic diseases remains inadequate in most regions of China. We identified several clinical and behavioral risk factors associated with worse functional outcomes in this population. This study serves as an important initial step toward multi-centered survivorship studies on psychosocial outcomes in the CCCG of China. Our study also supports the importance of educational initiatives by schools to meet the needs of children with chronic illnesses early during the recovery phase.

We did not expect late effects to be prevalent in the relatively young cohort of survivors who participated in this study, as they were an average of only 5 years post-diagnosis. However, half of the pediatric survivors had at least one parent-reported health problem. Those with multiple health problems had worse social and emotional functioning than those who did not indicate any health problems. This finding suggests that symptoms of preclinical health conditions affect various aspects of a child’s quality of life, even at the early stage of survivorship. Admittedly, the cross-sectional study design did not allow us to examine the temporal relationship among the health problems/ symptoms and psychosocial outcomes, and the assessment of current health problems/ symptoms was solely based on proxy reports from the parents. Although the responses were verified by further assessments from the clinicians, a more comprehensive assessment is necessary to verify these inferences and to interpret the findings in the light of clinical characteristics associated with health problems in this population. Nevertheless, our preliminary findings concur with evidence in the literature that cancer-related physical and psychological symptoms do co-exist as clusters [9,18,32], and that emerging health problems in young survivors are modifiable factors whose management can potentially improve survivors’ psychosocial functioning [43–45]. Taken together, our findings highlight that it is important to educate parents to monitor early symptoms of late effects in their children, and that parents must inform clinicians of these early symptoms during their routine LTFU visits with their children, so that late effects can be treated to minimize their adverse effect on children’s functional outcomes.

Almost a quarter of Chinese parents have indicated that it was “often true” that their child exhibited at least one behavioral problem on a child behavior scale. A study in the United States found that parent-reported impairments in school-aged survivors of pediatric leukemia, including depression, atypicality and attention problems, ranged from 17% to 26% [11]. As our study did not include age- and sex-matched controls, we were unable to assess if the rates of parent-reported behavioral problems in survivors differed from those in the general population. However, our findings are similar to one Chinese study, which reported that 40% to 78% of children who underwent HSCT experienced symptoms of obsession-compulsion, hostility, and interpersonal sensitivity [46]. Encouragingly, we also found that lower attention and behavioral problem scores were associated with a longer time since end of treatment, suggesting that these problems might resolve or decrease over time. Given the stigma associated with mental illness in Chinese culture, underdiagnosis or misdiagnosis of mental conditions in children is common in China [47,48]. Our future work includes conducting comprehensive behavioral assessment and performance-based neurocognitive tests to ascertain the prevalence of developmental and psychiatric disorders in pediatric cancer survivors. S assist clinicians to detect emerging disorders early, and thus provide support to alleviate the difficulties such children face when transitioning into young adulthood.

Under the healthcare system in China, pediatric patients with hematological malignancies or hematological disorders requiring HSCT are treated in different specialized centers from patients with certain solid tumors (e.g. CNS tumors). This might have resulted in a higher proportion of the children in the current study having hematological malignancies (66.2% of pediatric cancer survivors) than the overall proportion of hematological malignancy cases (44.1%) in China [49]. Survivors with hematological malignancies or non-CNS solid tumors are not as vulnerable to poor functioning as survivors with CNS tumors and those treated with cranial radiation [30,35]. This may explain why we did not observe differences between the outcomes of children with different diagnoses. Admittedly, our study lacked non-patient controls and we did not examine survivors’ comprehensive treatment records, such as their cumulative doses of chemotherapy, dosimetry data and on-therapy acute toxicities. It is also plausible that the lack of an association between treatment exposures and parent-reported behavioral symptoms suggests that treatment contributes less to the perceptions of their psychosocial well-being than their cancer experience (e.g. recurrent hospitalizations or outpatient visits due to long-term complications in patients with HSCT) [11,50]. In certain cancers or hematological disorders, HSCT may be considered as the only viable treatment option after treatment failures or disease relapse. Therefore, these children might suffer from poorer psychosocial outcomes as they tended to be sicker, have prolonged periods of hospitalization, and suffer from more severe chronic complications such as graft-versus-host disease [19,46]. Future work should also compare functioning in HSCT recipients treated with reduced intensive conditioning versus myeloablative conditioning as some groups have reported better quality of life in the former group [51,52].

Several behavioral correlates of psychosocial outcomes were identified in our study. First, pain interference was strongly associated with emotional distress and inattention. Other studies of children with chronic diseases have reported that pain with daily interference was associated with sedentary behavior, increased use of psychoactive medications and absenteeism from work/school [36,37,40]. We also identified sleep problems and excessive screen time as risk factors for poorer psychosocial outcomes. It may be possible that loneliness and poor social skills in these children might have led to excessive dependence on mobile technology and the internet [53,54]. Consistent with previous observations of school-aged children in China [55,56], psychological distress may also be triggered by the ongoing COVID-19 pandemic, as this has caused children to become more frequent users of computers and mobile technology, given that schools have been closed for extended periods. From a clinical perspective, oncology practitioners should consider implementing age-appropriate health behavior assessments throughout the survivorship trajectory of their patients to better capture the functional impact of these lifestyle factors.

This study has a few limitations. It comprised a relatively small number of children who were recruited from a single pediatric center in a relatively affluent urban city. Thus, it is unlikely to be a representative cohort as China is a geographically large region, with differences in resources and constraints between urban and rural areas [21]. Furthermore, our sample was heterogeneous in nature, as it consisted of children with multiple primary diagnoses. Our convenience sampling approach would also have missed survivors who were lost to follow-up from their primary pediatric clinics due to geographic distance and/or a lack of motivation to participate in surveillance care [56]. These study limitations could have introduced sampling bias and reduced the validity of our findings. Moreover, although the ACT questionnaire is typically used in clinical settings, it may have been insufficiently sensitive to capture differences between clinically relevant subgroups’ constructs of interest (e.g. treatment modalities) in our study population. A common source bias might account for the strong associations between parent-reported outcomes and behavioral factors. The lack of information on the parent respondent is another major limitation of this study. Their responses might also have reflected the psychological status of parents or their relationship with their child, rather than the child’s actual behavior [11,50]. This is especially relevant for the context of Asian societies, as our group has previously reported that parents’ age, educational attainment and beliefs might reflect proxy-reports of symptoms in Chinese children undergoing invasive procedures [57]. As there are now more validated and multilingual patient-reported outcome measures available for use in pediatric populations, future study designs should also include age-appropriate tools to capture pediatric survivors’ self-reported outcomes. There had been no pre-treatment testing of behavioral or cognitive symptoms in the children, as such assessments of young children are difficult to perform at the time of a cancer diagnosis. Finally, our study did not include a control group for comparison. Unlike other major cohorts in the United States and Europe [58,59], it is challenging for Chinese studies to recruit sibling controls due to the long-lasting “one-child policy” that was only abolished recently in 2015 [60]. Nevertheless, our study sheds light on the potential psychosocial burden experienced by pediatric survivors of cancer and hematological disorders. A multicenter study that involves prospective LTFU patients may better reflect the trajectory and temporal association between covariates and functional outcomes as these patients transition from early post-treatment into long-term survivorship.

Notwithstanding the above limitations, our study provides preliminary evidence on the relationship between modifiable risk factors and psychosocial outcomes in pediatric survivors of cancer and bone marrow disorders. The continued development of national and international collaborative initiatives is expected to yield further advances in the treatment of children with such catastrophic diseases. As such, recent efforts have been geared toward improving the quality of care for the emerging population of children with chronic diseases in China. For example, in 2019, expert panelists representing major institutions within the CCCG developed a framework for establishing collaborative strategies to facilitate follow-up care of pediatric cancer survivors in China [21]. A high-priority in this framework is to explore the feasibility of implementing cognitive and psychosocial screening strategies in community settings. The approach would leverage support from primary care practitioners and nongovernmental organizations to provide psychosocial support and conduct basic screening services at sites that are easily accessible to survivors when they return to school after treatment.

## Conclusion

Our results show that subgroups of pediatric survivors of cancer or bone marrow failure disorders in our sample experienced poorer psychosocial outcomes. Survivors who were diagnosed at a younger age or had unaddressed/untreated symptoms or health problems may therefore require additional screening for psychological distress. Rehabilitation interventions should address modifiable behavioral factors, such as sleep habits, screen time and chronic pain. Our future includes conducting a multicenter study to validate our findings in a larger sample of survivors in mainland China. The implementation of psychosocial assessments during routine LTFU care may help to identify high-risk patients during the early phase of survivorship. Educational needs and functional limitations should also be evaluated prospectively in this population.

## Supporting information

S1 File Supplement 1: Rates of parent-reported health problems or symptoms in survivors. (DOCX)

S2 File Supplement 2: Exploratory analysis of psychosocial outcomes in survivors by types of health problems. (DOCX)

## Abbreviations

ACT: After Completion of Therapy
ADHD: Attention deficit hyperactivity disorder
CCCG: Chinese Children’s Cancer Group
CNS: Central nervous system
Est: Estimates
HSCT: hematopoietic stem cell transplantation
LTFU: Long-Term Follow-up
SCMC: Shanghai Children’s Medical Center
SD: Standard deviation
SE: Standard errors
