# Supplementary material for: Psychosocial outcomes in Chinese survivors of pediatric cancers or bone marrow failure disorders: A single-center study
Source: PLoS One. 2022 Dec 13;17(12):e0279112. doi: 10.1371/journal.pone.0279112 (PMC9746993; doi:10.1371/journal.pone.0279112)
Supplement: S1 File — (DOCX) [file pone.0279112.s001.docx]

**SUPPLEMENT 1 Rates of parent-reported health problems or symptoms in survivors**


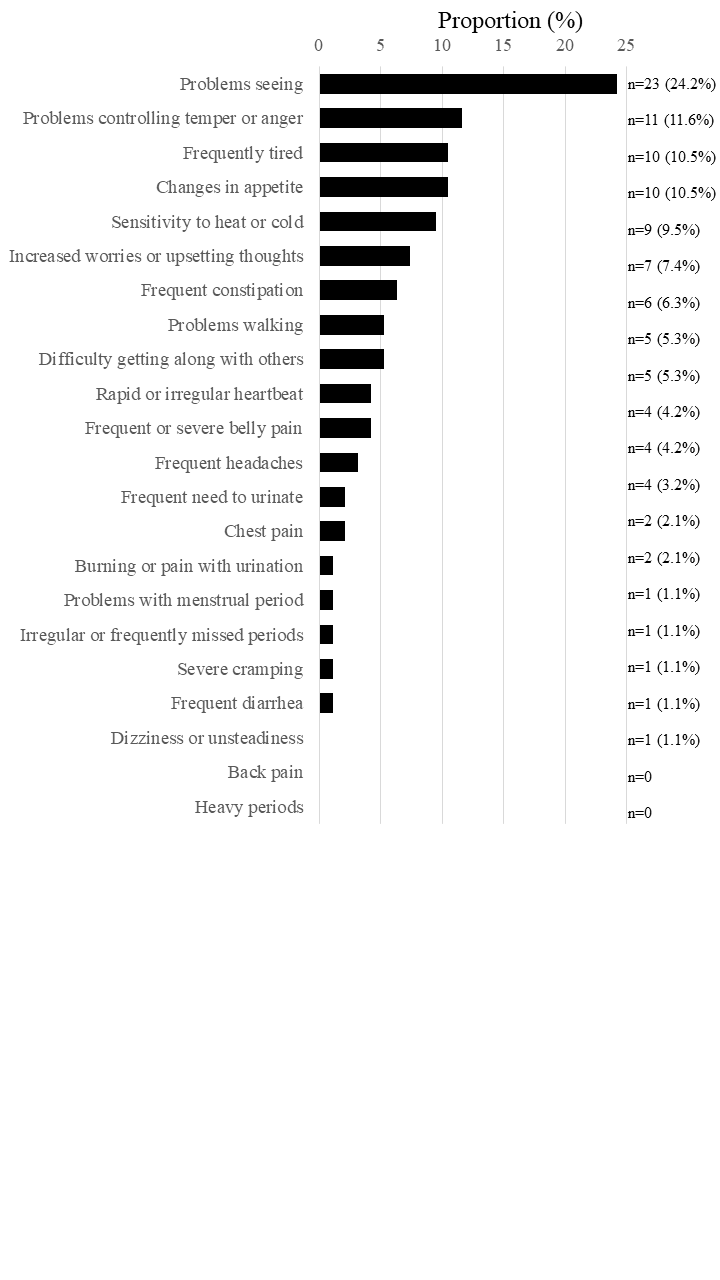


Black bar: Refers to participants who indicated “currently present” for the specific health problem/ symptom
