# Supplementary material for: Psychosocial outcomes in Chinese survivors of pediatric cancers or bone marrow failure disorders: A single-center study
Source: PLoS One. 2022 Dec 13;17(12):e0279112. doi: 10.1371/journal.pone.0279112 (PMC9746993; doi:10.1371/journal.pone.0279112)
Supplement: S2 File — (DOCX) [file pone.0279112.s002.docx]

**SUPPLEMENT 2:** **Exploratory analysis of psychosocial outcomes in survivors by types of health problems**

|  | Psychosocial Impairment Scores* | | | |
| --- | --- | --- | --- | --- |
|  | **Social functioning** | **Emotional functioning** | **Attention/ Concentration** | **Child behavior** |
| Score range | [1 – 35] | [1 – 30] | [1 – 30] | [1 – 69] |
| All survivors | 13.9 ± 5.6 | 8.8 ± 3.5 | 13.7 ± 5.0 | 29.6 ± 7.5 |
| Types of health problems |  |  |  |  |
|  | ***P=* 0.02** | *P=* 0.23 | *P=* 0.06 | *P=* 0.19 |
| Presence of health problems/ symptoms related to psychosocial functioning^ | 15.9 ± 5.7 | 12.4 ± 4.6 | 15.8 ± 3.4 | 30.0 ± 9.1 |
| Presence of other health problems/ symptoms | 12.9 ± 5.2 | 11.6 ± 5.3 | 14.6 ± 5.5 | 33.0 ± 8.1 |
| No current health problems/ symptoms | 12.4 ± 5.2 | 11.2 ± 5.3 | 12.5 ± 4.7 | 28.3 ± 6.2 |

* A higher score is indicative of worse functioning

^ Health problems or symptoms related psychosocial or/and cognitive functioning, based on existing literature (chronic or persistent pain, cardiac symptoms, and fatigue) [7,9,10,32,36,40-42]. For this study, the specific health problems or symptoms referred to “Frequent headache” and “Frequent or severe belly pain” (chronic or persistent pain), “Frequently tired” (fatigue), and “Rapid or irregular heartbeat” and “Chest pain” (cardiac symptoms).
